# LOW RESILIENCE FACTORS AND MINORITY RACE PREDICT QUALITY-OF-LIFE DEFICITS AMONG OLDER ADULTS WITH CHRONIC DISEASE

**DOI:** 10.1093/geroni/igad104.3043

**Published:** 2023-12-21

**Authors:** Allan Nkwata, Xiao Song, Ming Zhang, Amara Ezeamama

**Affiliations:** University of Michigan, Ann Arbor, Michigan, United States; University of Georgia, Athens, Georgia, United States; University of Georgia, Athens, Georgia, United States; Michigan State University, East Lansing, Michigan, United States

## Abstract

Resilience-promoting factors (RPF), minority race and their interaction, were evaluated as determinants of quality of life (QOL) decline in a nationally representative sample of US adults ≥50 years old (N=3932) with heart disease and/or type-2 diabetes from 2006-2014 as part of the Health and Retirement Study. RPF included personal mastery and positive social support (PSS). QOL was assessed by self-rated health (SRH) and defined as poor (fair/poor), good, or excellent (very good/ excellent). Repeated measures multinomial regression using generalized estimating equations (GEEs) related RPF, race, and their interaction to SRH declines over 8 years. Odds ratios (OR) and 95% confidence intervals (CIs) were calculated with adjustment for time, sociodemographic and behavioral confounders. Low mastery, low PSS and minority race were each associated with higher odds of QOL declines over 8 years (mastery OR- 2.27, 95% CI: 1.83- 2.81; PSS OR- 1.35, 95% CI: 1.10-1.65; African American OR- 1.46, 95% CI: 1.25-1.70, and Other race OR- 1.43, 95% CI: 1.10-1.86). QOL declines related to PSS-friends varied by race and time (race x time x PSS-friends, p=0.093). Among older Caucasians, the association between PSS and QOL declines did not vary over time (p=0.676). However, among African Americans and Other race, low PSS was associated with increased odds of QOL declines over time (p=0.031 and p=0.034 respectively). RPF and minority race predicted QOL decline in older Americans with comorbid conditions. Policies/ interventions to enhance resiliency represent a viable strategy for mitigating racial disparities in overall wellbeing and improving health outcomes in aging Americans.

